# Deciphering of the Human Interferon-Regulated Proteome by Mass Spectrometry-Based Quantitative Analysis Reveals Extent and Dynamics of Protein Induction and Repression

**DOI:** 10.3389/fimmu.2017.01139

**Published:** 2017-09-14

**Authors:** Dominik A. Megger, Jos Philipp, Vu Thuy Khanh Le-Trilling, Barbara Sitek, Mirko Trilling

**Affiliations:** ^1^Medizinisches Proteom-Center, Ruhr-Universität Bochum, Bochum, Germany; ^2^Institute for Virology, University Hospital Essen, University Duisburg-Essen, Essen, Germany

**Keywords:** interferon, IFN-stimulated gene, IFNalpha, IFNgamma, mass spectrometry, proteome, IFN-repressed gene

## Abstract

Interferons (IFNs) are pleotropic cytokines secreted upon encounter of pathogens and tumors. Applying their antipathogenic, antiproliferative, and immune stimulatory capacities, recombinant IFNs are frequently prescribed as drugs to treat different diseases. IFNs act by changing the gene expression profile of cells. Due to characteristics such as rapid gene induction and signaling, IFNs also represent prototypical model systems for various aspects of biomedical research (e.g., signal transduction). In regard to the signaling and activated promoters, IFNs can be subdivided into two groups. Here, alterations of the cellular proteome of human cells treated with IFNα and IFNγ were elucidated in a time-resolved manner by quantitative proteome analysis. The majority of protein regulations were strongly IFN type and time dependent. In addition to the expected upregulation of IFN-responsive proteins, an astonishing number of proteins became profoundly repressed especially by IFNγ. Thus, our comprehensive analysis revealed important insights into the human IFN-regulated proteome and its dynamics of protein induction and repression. Interestingly, the new class of IFN-repressed genes comprises known host factors for highly relevant pathogens such as HIV, dengue virus, and hepatitis C virus.

## Introduction

Interferons (IFNs) are pleiotropic cytokines, which are rapidly expressed upon encounter of pathogens such as viruses, bacteria, and fungi or in the presence of tumors. Mutations impairing the ability to stimulate IFN secretion or to recognize and adequately respond to IFNs have drastic consequences in terms of exaggerated pathogen susceptibility and increased tumor frequencies. Mice harboring targeted mutations in central components of the IFN system succumb to experimental infections with various pathogens even at very low doses of infection (sometimes in the range of the respective detection limit or even below), while wild-type animals easily survive infections with high numbers of the same pathogen (1, 2). Human individuals suffering from similar mutations have been identified—often due to overt morbidity and mortality after infection with attenuated live vaccine viruses or otherwise mild and/or opportunistic agents (3). The importance of IFNs in control of tumors is evident from the findings that mice lacking functional IFN systems are more prone to spontaneous tumor development and increased tumor burden in experimental models (4–6) as well as from the fact that loss-of-function mutations become enriched in genes coding for central mediators of the IFN system during tumor development (e.g., in the case of melanoma) (7, 8) indicating a pronounced selection pressure elicited by the IFN system.

Interferons influence numerous fundamental biological processes such as cell proliferation and protein translation. Consistently, the expression of IFNs has profound consequences and must be controlled tightly. On the organism level, IFN treatment is often associated with flu-like symptoms and can cause significant side effects (e.g., depression). Excessive IFN induction and/or signaling due to mutations can result in diseases called interferonopathies (9).

According to their molecular homology and their receptor usage, IFNs can be subdivided into type I, type II, and the recently described type III IFNs. Type I IFNs are comprised of all IFNα subclasses and IFNβ. IFNγ is the only member of type II IFN (IFN-II). The family of type III IFNs comprises several IFNλ subtypes. Different recombinant IFNs have been or are currently in use as drugs. Human IFNα2A and α2B, a combination of IFNα2A, B, and C, as well as a synthetic designer molecule based on the consensus of different IFNα subtypes, IFNβ and IFNγ have been approved by the FDA to treat different infectious diseases (see http://www.accessdata.fda.gov/scripts/cder/daf/index.cfm). However, the most frequently prescribed IFN is IFNα2.

All type I IFNs (IFN-I) bind to the same receptor complex composed of IFNAR1 and IFNAR2 which are preassociated with tyrosine kinase 2 (Tyk2) and Janus kinase 1 (Jak1), respectively. Upon binding of the IFN-I to their cognate receptor complex, the Janus kinases phosphorylate the intracellular domains of the receptor chains thereby generating binding sites for signal transducer and activator of transcription (STAT) 1 and STAT2. After the STATs bound the receptors, they become phosphorylated by Tyk2 and Jak1 at a specific tyrosine residue located around amino acid position 700 (Y701 in the case of STAT1). Due to intramolecular interactions between the phosphorylated tyrosine residue of one STAT molecule with the src homology 2 domain of the second STAT molecule and *vice versa*, an active heterodimer forms. Previous models often implied a *de novo* interaction of monomeric STAT proteins upon phosphorylation, whereas recent work argues in favor of preformed STAT dimers (10, 11) which change their conformation and orientation upon activation (12–14). Together with IFN regulatory factor 9 (IRF9), STAT1, and STAT2 form active heterotrimers called IFN-stimulated gene factor 3 (ISGF3) which translocate into the nucleus, bind to specific DNA enhancer elements [called IFN-stimulated response elements (ISREs)] and induce the expression of adjacent genes. The binding and recognition of DNA is mediated by both STATs and the IRF9 molecule. Consistently, the central part of the ISRE consensus resembles an IRF DNA binding site (also called IRF-E site) (15).

Type III IFNs have been shown to bind to a distinct receptor complex which is only expressed in certain tissues, but to induce an IFN-I-like signaling and thereby a similar transcriptional ISRE response (16, 17). Hence, type I and type III IFNs can be grouped according to the activation of ISGF3 transcription factor complexes inducing genes harboring ISRE promoter/enhancer elements.

Interferon γ binds to a receptor composed of IFNGR1 and IFNGR2 which are preassociated with Jak1 and Jak2, respectively. In clear contrast to IFN-I and IFN-III, IFNγ mainly induces STAT1 homodimers. Since the recognition of DNA relies on STAT1 molecules (and not on the IRF molecule IRF9), the respective DNA enhancer element, called gamma-activated sequence (GAS), represents a canonical STAT DNA-binding site and differs from ISRE elements. IFN responsiveness of a given gene is considered to be defined by the presence as well as the number, distance, and arrangement of ISRE and GAS enhancer elements.

Beside this two canonical signaling pathways (activating ISRE and GAS) separating type I and III IFNs from type II IFNs, several non-canonical signaling events have been described: for example, IFN-I induce STAT1 homodimers (in this case called alpha activated factor) which elicit an IFNγ-like response (18) and IFNγ induces STAT2 and IRF9 containing complexes which stimulate ISRE-like responses (19–22). Beneath this receptor proximal signaling events, cross-talk can also be induced down-stream by induction of a second layer of transcription factors (e.g., IRFs): IFNγ strongly induces IRF1 which in turn can enhance genes harboring IRF-E sites. Since the central part of ISRE elements resembles an IRF-E site, IFNγ can stimulate several IFN-I responsive genes indirectly *via* IRFs like IRF1. Given these descriptions of overlap and crosstalk between the signaling cascades, it is surprising that both cytokines are thought to induce different biological responses: IFN-I (and IFN-III) are believed to induce a direct antiviral activity, whereas IFNγ is mainly considered as stimulator of adaptive immune responses (e.g., by enhancing antigen presentation).

A great wealth of IFN-stimulated genes (ISGs) have been described in the past using various techniques. However, we and others have provided evidence that especially IFNγ can also repress the transcription of a considerable number of genes, which we termed IFN-repressed genes (IRepGs) (22). This finding raises the apparent and relevant question if such a regulation results in an altered protein composition of IFN-exposed cells—especially in humans.

Here, we applied label-free quantification based on mass spectrometry to determine amplitude and dynamics of IFN-induced changes in the human proteome. We analyzed dynamic changes elicited by IFNγ in comparison to ISRE-activating IFNs. Since IFN-I and IFN-III activate similar signaling cascades, we focused on IFNα2 as archetypical IFN due to its prominent relevance as antiviral drug. For consistency and reproducibility, we chose a diploid human cell line which has been used extensively to generate several vaccines and to propagate several viruses, lacks neoplastic properties (23), and which has been shown to be reprogrammable by forced expression of pluripotency inducing transcription factors (24) as a hallmark of retained nativeness.

## Materials and Methods

### Cell Culture

Human MRC-5 cells were obtained from American Type Culture Collection and cultured in six-well plates with Dulbecco’s modified Eagle’s medium supplemented with 10% (v/v) fetal calf serum (FCS) and 100 U/mL penicillin and 100 µg/mL streptomycin. The treatment with 500 U/mL IFN-α and IFN-γ (Hu-IFN-α2a, Hu-IFN-γ; PBL Assay Science) containing media started after cells were washed with PBS. Cells were subsequently incubated for 4, 24, and 48 h, respectively, at 37°C and 5% CO_2_.

### Cell Harvesting and Lysis

Cells were harvested and washed twice with ice-cold sterile PBS [4°C, 2 min, 4,000 rpm (1,699 rcf)], suspended and lysed in 50 mM triethylammoniumbicarbonat containing 0.1% (w/v) Rapigest SF Surfactant (Waters). After 3 min of sonication, cell suspensions were centrifuged for 40 min at 4°C and 12,700 rpm (18,213 rcf) to remove cell debris. Supernatant was stored at −80°C until analysis.

### Digestion Protocol

For each sample, a protein amount of 4 µg was reduced using 20 mM dithiothreitol for 30 min at 60°C and alkylated with 15 mM iodoacetamide for 30 min at room temperature. Trypsin was added (3 µL, *c* = 0.1 µg/µL) for digestion for 16 h at 37°C. Enzyme activity was quenched by acidification using 10% (v/v) of TFA for 30 min at 37°C. Insoluble hydrolyzed surfactant was removed by 10 min centrifugation at 14,000 *g*. The supernatant was collected and dried in a centrifugal evaporator. 300 ng of each sample were pooled to obtain a master mix used for the monitoring of LC performance during the whole experiment and the alignment of LC-MS/MS runs in the subsequent quantitative analysis.

### LC-MS/MS Analysis

For an unbiased analysis, samples derived from different experimental groups were analyzed in a randomized fashion. For each measurement, 300 ng of tryptic peptides were dissolved in 15 µL 0.1% (v/v) TFA and injected into an Ultimate^®^ 3000 RSLC nanoLC system online coupled to an Orbitrap Elite mass spectrometer (both Thermo Scientific). The peptides were pre-concentrated for 7 min on a trap column (Acclaim^®^ PepMap 100, 75 µm × 2 cm, C18, 5 µm particle size, 100 Å pore size) using 30 µL/min 0.1% (v/v) TFA and subsequently separated on an analytical column (Acclaim^®^ PepMap RSLC, 75 µm × 50 cm, nano Viper, C18, 5 µm particle size, 100 Å pore size) by applying a gradient from 5 to 40% solvent B over 98 min [solvent A: 0.1% (v/v) formic acid; solvent B: 0.1% (v/v) formic acid, 84% acetonitrile; 400 nL/min; column oven temperature 60°C]. Full scans were acquired in the Orbitrap analyzer with a resolution of 60,000 in a data-dependent mode. The 20 most abundant ions of a spectrum acquired at MS1 level were fragmented by collision-induced dissociation and measured in the linear ion trap.

### Data Analysis

Peptide identification was conducted using Proteome Discoverer 1.4 software (Thermo Scientific, Bremen, Germany). Database search was performed with Mascot (v. 2.5.1, Matrix Sciences Ltd., London, UK) against the UniProt-SwissProt database (Release 2014_10; v. 2.5; 546,790 sequences). Taxonomy was restricted to *Homo sapiens* (20,194 sequences). Trypsin was set as cleaving enzyme with one allowed missed cleavage site. Mass area was set to 350–10,000 Da and mass tolerances to 5 ppm for the precursor and 0.4 Da for fragment ions, respectively. Oxidation of methionine was set as dynamic modification and carbamidomethylation of cysteine as static. Confidence of peptide identification was assessed using the Target Decoy PSM Validator node implemented in Proteome Discoverer. Peptide identifications with false discovery rate (FDR) <1% were considered. Protein grouping function was applied.

The quantitative data analysis was performed using Progenesis QI for proteomics (v. 2.0.5387.52102; Non-linear Dynamics, Newcastle upon Tyne, UK). Briefly, LC-MS/MS runs were imported and aligned to a master mix run. During feature detection, only signals with at least three isotopes and charges of +2 to +5 were considered. After deleting features not satisfying the mentioned criteria, raw abundances of the features were normalized for correcting experimental variations (25). In the subsequent step, LC-MS/MS runs exhibiting normalization factors between 0.5 and 2.0 were considered for further analysis and grouped according to the experimental groups. Identifications of peptides and proteins obtained were then mapped to the respective features by importing the result files from Proteome Discoverer. Protein quantification was conducted using non-conflicting peptides only and protein grouping option was disabled.

Proteomics data have been deposited as complete submission to the ProteomeXchange Consortium (http://proteomecentral.proteomexchange.org) *via* the PRIDE partner repository with the data set identifier PXD006442 and DOI 10.6019/PXD006442. Data were uploaded using the ProteomeXchange Submission Tool (ver. 2.3.2). ProCon—PROteomics CONversion tool (ver. 0.9.641) was used for the necessary conversion of Proteome Discoverer result files into the mzIdentML standard format (26).

Functional annotations were performed using the Database for Annotation, Visualization, and Integrated Discovery (DAVID, ver. 6.8) (27, 28).

### Statistical Analysis

Normalized protein abundances were exported from Progenesis QI and arcsinh-transformed. Using an in-house written R script (R Foundation for Statistical Computing, Vienna, Austria). Benjamini–Hochberg corrected one-way ANOVA was used for the calculation of the FDR-corrected *p*-values (29). For proteins passing a significance level of 0.05, a *post hoc* test (Tukey’s honest significant difference method) was conducted to obtain *p*-values for pairwise comparisons.

### Immunoblots

Immunoblots were performed as described previously (30).

### Flow Cytometry

The flow cytometric analysis was performed as described previously (31) using herein indicated antibodies.

## Results

### IFNs Alter the Human Proteome by Protein Induction and Repression

**Figure 1:**
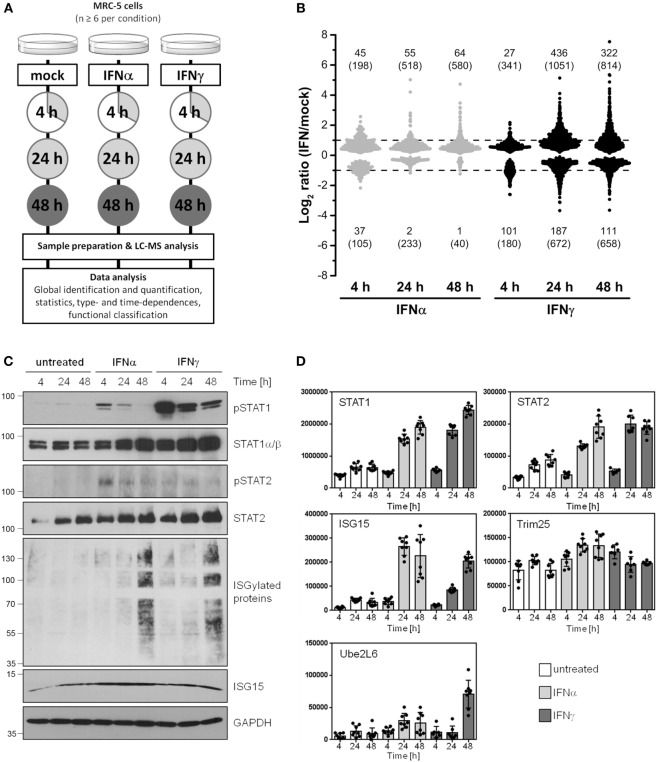
Global identification and quantification of interferon (IFN)-regulated proteins in human cells. **(A)** General design of the conducted proteomics study. **(B)** Overview of significantly regulated proteins (*p* < 0.05) depending on the IFN type (IFNα: gray, IFNγ: black) and exposure times (4, 24, and 48 h). Fold change thresholds (twofold regulation) are indicated as dashed lines. The number of proteins passing the significance threshold is shown in brackets. The numbers of proteins satisfying significance as well as fold change criteria are indicated without brackets. **(C)** Experimental conditions as in **(A)**, but cells were harvested and lysed for immunoblot analysis using indicated antibodies. The lines and numbers on the left depict the migration of the marker proteins with indicated molecular weights in kDa. **(D)** The normalized abundances of the same proteins (or their regulators) shown in **(C)** calculated by use of the MS data are depicted. As in all analyses, unstimulated controls are depicted in white, IFNα stimulation in light gray, and IFNγ stimulation in dark gray bars, respectively. Individual quantifications (*n* = 6–8) are indicated as dots, bars depict mean values with SD (error bars).

According to the study design shown in Figure 1A, the proteome alterations in human cells induced by IFNα and IFNγ were analyzed by means of ion-intensity-based label-free quantitative proteomics. To elucidate time-dependent effects, protein abundance changes in at least six biological replicates relative to mock-treated control cells were monitored after 4, 24, and 48 h.

In the whole LC-MS/MS study, 2,945 proteins were successfully identified and quantified with at least one unique peptide in 69 parallel samples (see Table S1 in Supplementary Material). A brief inspection of the corresponding names of the proteins most significantly upregulated by IFN (e.g., IFN-induced GTP-binding protein Mx2) immediately highlighted an enrichment of IFN-stimulated proteins indicating the validity of our approach. To cope with the issue of less accurate quantification results based on single peptide quantifications (32, 33), we determined individual coefficients of variation (CVs) for each protein represented by a single unique peptide. Single peptide quantifications with large deviations indicated by an averaged CV >50% were excluded. A comparison of the median CVs showed that this revised group of proteins with single peptide quantification exhibits the same variation as observed for proteins quantified with two peptides (see Figure S1 in Supplementary Material). The number of proteins finally considered for the data analysis concerning time- and type-dependent proteome alterations was 2,735 (see Supplementary Material for the complete data set).

After quantitative and statistical analyses, the number of proteins showing a significantly different abundance (*p* < 0.05) in IFN-treated samples in comparison to mock-treated controls was determined for each IFN type and incubation time. Depending on the IFN type and duration of treatment, the abundance of up to 1,700 proteins significantly changed (see number in brackets in Figure 1B). Based on our experience with the limitations of label-free quantification (34) and our criteria of biological significance and meaningfulness, we set a threshold of twofold change in terms of median protein abundance (see numbers without brackets in Figure 1B) for subsequent analysis. Since changes below this rather arbitrarily chosen set point might be relevant under particular circumstances, these proteins were included in the Supplementary Material enabling others to reanalyze the data with higher or lower criteria of stringency.

Depending on the IFN type and the period of exposure, more than 600 proteins significantly changed their abundance more than twofold, highlighting profound alterations of the cellular protein composition in response to IFN. After 4 h of IFN exposure, IFNα treatment led to a higher number of proteins being upregulated when compared with IFNγ (45 versus 27 proteins). In clear contrast, after 24 and 48 h, the number of upregulated proteins is five to eight times higher in the case of IFNγ (55 versus 436 at 24 h). We also observed several proteins which were significantly downregulated. In this respect, the difference between IFNα and IFNγ was even more pronounced, since such protein regulations were clearly more prominent upon IFNγ treatment. Functional annotations of all proteins being significantly and at least twofold regulated by IFNα or IFNγ were performed for each investigated time point. The obtained results display a broad spectrum of associated molecular functions, biological processes and cellular components (see Figures S2–S4 in Supplementary Material). As expected, for both IFN types, IFN signaling (and several related or similar gene ontologies called, e.g., “defense response to virus”) was found as a highly enriched biological process among the upregulated proteins. Additionally, several biological processes associated with antigen presentation *via* MHC-I were found to be enriched among proteins induced by both IFNs after prolonged exposure. In the particular case of IFNγ, the obtained results furthermore indicate, that a broad spectrum of diverse biological processes are altered. This is in line with the expectations based on the tremendously higher number of proteins being regulated by IFNγ when compared with IFNα. In addition, the dynamic nature of cellular proteome modulations is nicely demonstrated by the enrichment of particular biological processes among up- and downregulated proteins at various time points. For example, different translational processes were found highly enriched among IFNγ-repressed proteins at 24 h, whereas the same processes were enriched in the IFNγ-induced group of proteins at 48 h (see Supplementary Material).

### Validation of Proteomic Changes Observed by Mass Spectrometry

To ensure appropriate IFN stimulation conditions and to further validate our LC-MS-based results, we prepared lysates and subjected them to SDS PAGE and subsequent immunoblot analysis. Membranes were probed with antibodies specific for tyrosine phosphorylated (“active”) STAT1 and STAT2 molecules as well as for STAT1, STAT2, and ISG15. As expected, IFN-treatment induced STAT1 and STAT2 phosphorylation and led to an increase of the IFN-responsive gene ISG15 as well as ISG15-conjugated proteins (Figure 1C). Consistent with the notion that STAT1 and STAT2 are IFN-responsive themselves [e.g., Ref. (35) and others], both proteins became also upregulated during IFNα and IFNγ exposure. The results obtained by LC-MS-based quantification were consistent with the results obtained by immunoblot (Figure 1D). Due to their prominent role in protein ISGlation (36, 37), quantification data for Trim25 (also called EFP) and Ube2L6 (also called UBCH8) are also depicted (Figure 1D).

### Type I and Type II IFNs Induce Discrete Changes in the Human Proteome

**Figure 2:**
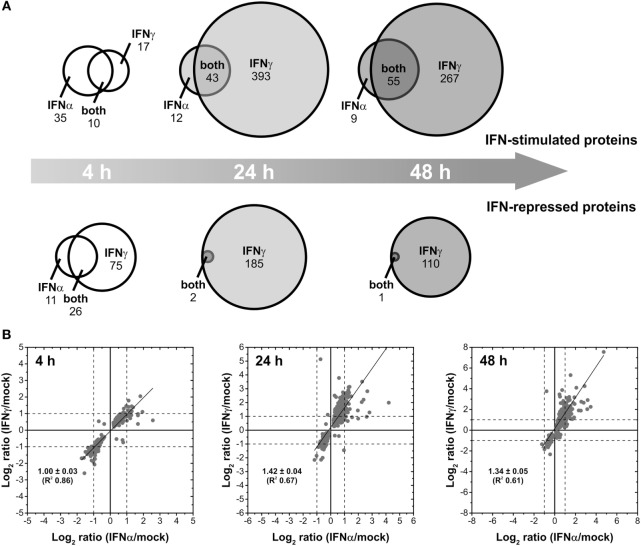
Differential changes of the proteome induced by different interferons (IFNs). **(A)** Venn diagrams illustrating the direct comparison of the number of IFNα- and IFNγ-stimulated and repressed proteins (*p* < 0.05, at least twofold regulation) after different times of exposure. **(B)** Scatter plots showing the linear correlation of IFNα- and IFNγ-induced protein regulations (log_2_-transformed ratios of significant regulations with *p* < 0.05). The calculation of the regression lines reveals stronger IFNγ-induced regulations after prolonged exposure (24 and 48 h).

Interferons differ concerning their biological responses. Depending on the nature of the respective pathogen, prominent discrepancies concerning the direct antiviral activity have been documented [e.g., (38)]. According to an oversimplified textbook concept, IFNα acts directly antiviral (e.g., by inducing effector proteins such as Mx, PKR, and OAS), whereas IFNγ modulates adaptive immune responses for example by stimulating MHC expression. Such differences should be reflected by non-overlapping changes in the proteome. Therefore, IFN type-specific complementarities were analyzed on qualitative level to elucidate to which extent both IFNs regulate common as well as distinct sets of proteins. To this end, lists of proteins exhibiting significant at least twofold up- and downregulations after treatment with IFNα and IFNγ, respectively, were compared for each time point of analysis. The results are shown in Figure 2A. After a short incubation time of 4 h, IFNα and IFNγ had only 10 upregulated and 26 repressed proteins in common. However, 17 and 35 proteins were exclusively upregulated by IFNγ and IFNα, respectively. Similarly, 75 proteins were only repressed by IFNγ and 11 proteins only by IFNα after 4 h of treatment. At later time points, the majority of proteins being IFNα responsive were also responsive to IFNγ. On top of these commonly responsive proteins, IFNγ was capable to specifically upregulate additional proteins (393 and 267) after 24 and 48 h of treatment. Concerning downregulated proteins, this trend was even more pronounced: after 24 and 48 h, only 2 and 1 protein, respectively, were significantly and at least twofold repressed by IFNα, whereas IFNγ also repressed these proteins plus 185 and 110 additional proteins, respectively (Figure 2A).

Beside this qualitative assessment, our approach also allows direct quantitative comparisons of IFNα- and IFNγ-induced changes by means of correlation analyses. Therefore, we compared the strength of regulation by IFNα in relation to the strength of regulation by IFNγ in scatter plots (Figure 2B). In contrast to the previous analysis, all proteins showing significantly altered abundances upon IFN-treatment were included irrespective of the fold change. Linear regression analyses for the investigated time points are shown in Figure 2B. For each time point, proteins being at least twofold regulated by both IFN types showed identical regulation direction—as indicated by the apparent lack of proteins in quadrants II and IV (Figure 2B). With only few exceptions, most proteins being specifically regulated at least twofold by one IFN type exhibit a consistent and significant trend upon treatment with the other IFN. However, in the latter case, fold change criteria are often not fulfilled. Regarding the strength of regulations, at 4 h posttreatment the slope of the regression line (~1) indicates that both IFNα and IFNγ have comparable inductive or repressive effects. As indicated by slopes above 1, at 24 and 48 h, protein regulations induced by IFNγ are more pronounced than the corresponding regulations by IFNα.

### Proteomic Changes Induced by IFNs are Highly Dynamic

**Figure 3:**
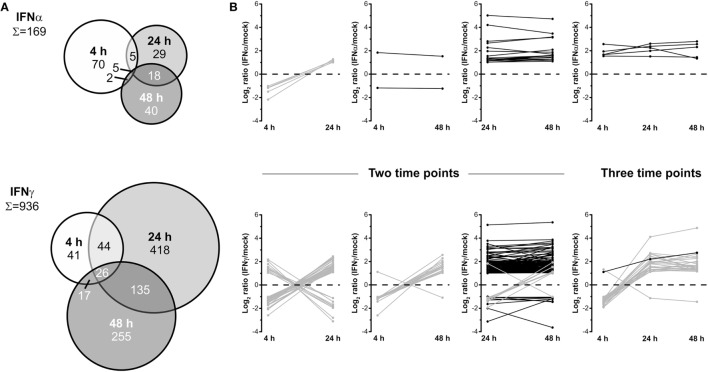
Dynamics of the human interferon (IFN) proteome. **(A)** Venn diagrams illustrating the direct comparison of numbers of significantly and at least twofold regulated proteins after different treatment times. **(B)** Regulation profiles (log_2_-transformed ratios) for proteins being significantly and at least twofold regulated at more than one individual time point. Proteins showing inconsistent regulations at different time points are shown in gray and those consistently up- or downregulated in black.

To elucidate time-dependent proteome alterations observed for a single IFN type, proteins significantly regulated at least twofold at 4, 24, and 48 h posttreatment were compared. The results of these accession-based comparisons are depicted as Venn diagrams in Figure 3A. In the case of both IFNs, proteome alterations are found to be highly dynamic as indicated by distinct complementarities between the investigated time points. For IFNα, 169 proteins were found to be significantly regulated at least at one of the three investigated time points. Of these, only 2, 5, and 18 proteins were regulated at two time points and only 5 at each time point after IFNα treatment. A closer inspection of these proteins (Figure 3B) revealed common regulation directions throughout the investigated time period, except contrary regulations of five proteins being significantly regulated at 4 and 24 h. Conversely, 70, 29, and 40 proteins were only differentially regulated at one particular time point. In the case of IFNγ, of 936 significantly at least twofold regulated proteins, 17, 44, and 135 proteins were regulated at two and 26 at all three time points. Forty-one, 418, and 255 proteins were only regulated by IFNγ at one particular time point. Interestingly, except IFIT3, all proteins found in the overlaps with the 4 h samples show inverted regulation profiles at one or both of the two other time points. Contrarily, of 135 proteins commonly altered after 24 and 48 h, 127 exhibit the same regulation direction (Figure 3B lower panel). Taken together, these data reveal an extraordinary dynamic and turnover of IFN-induced changes and highlight the necessity to perform such experiments in a time-resolved manner—otherwise considerable changes might be missed or at least severely underestimated.

### Type I IFNs Modulate the Antigen Presentation Machinery

**Figure 4:**
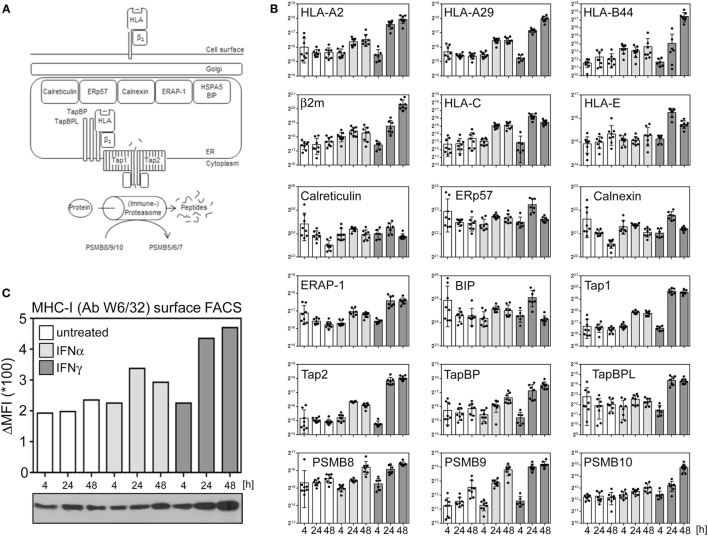
Interferon (IFN) responsiveness of the components of peptide loading and antigen MHC presentation. **(A)** Simplified schema of peptide loading and MHC/human leukocyte antigen (HLA) presentation. See text for more details. **(B)** Normalized abundances of indicated proteins at indicated time points. The depicted proteins were selected based on their well-known role in peptide generation, peptide loading and/or MHC presentation. Unstimulated controls are depicted in white, IFNα stimulation in light gray, and IFNγ stimulation is indicated in dark gray bars, respectively. Individual quantifications (*n* = 6–8) are depicted as dots, bars indicate mean values with SD (error bars). **(C)** MHC-I surface disposition determined by flow cytometric analysis using the W6/32 antibody which recognizes β2m-associated HLA-A, -B, and -C molecules (upper bar chart) and overall protein abundance of the HLA heavy chains as determined by immunoblot using the HC10 antibody (lower panel).

Nucleated cells continuously present a snapshot of their current protein expression profile in the context of human leukocyte antigen (HLA) molecules to circulating T lymphocytes (see Figure 4A for an overview). Especially IFNγ is well known for its effect on several proteins implicated in antigen presentation. The proteasome and other proteases degrade proteins to peptides which are transferred into the lumen of the ER by the transporter associated with antigen presentation (TAP). Via TabBP (also called Tapasin), TAP is associated with HLA molecules, composed of the HLA heavy chain and β2m. The loading of peptides onto HLA/MHC molecules as well as the quality control are catalyzed by several chaperons (e.g., ERp57, calreticulin, calnexin). Increased expression of several genes involved in antigen presentation induced by IFNγ is well described. Additionally, IFNγ induces a change in the composition of the proteasome from the constitutive proteasome to the so called immune proteasome by stimulating an exchange of three subunits (PSMB5, 6, and 7) by three alternative subunits [namely PSBM8 (LMP7), 9 (LMP2), and 10 (MECL1)]. Based on the proteome data, we exemplarily compared the regulation of proteins implicated in HLA presentation after IFNα and IFNγ treatment. Consistent with previous reports about MRC-5 cells (39), our proteome analysis identified and quantified HLA proteins corresponding to HLA-A2, -A29, and B44 alleles. Although IFNγ was superior in inducing the components of antigen presentation, IFNα also enhanced the abundance of several proteins (e.g., HLA-A, HLA-B, HLA-C, PSBM9, TAP1, and TAP2) (Figure 4B). Consistent with this IFNα responsiveness of important components of the HLA/MHC presentation pathway, a flow cytometry experiment using the HLA-specific antibody W6/32 showed an upregulation of HLA presentation on the cell surface of IFNα- and IFNγ-treated cells (Figure 4C) validating our proteome data on biological level.

### Type II IFN Upregulates a Specific Set of Antiviral Effector Proteins

**Figure 5:**
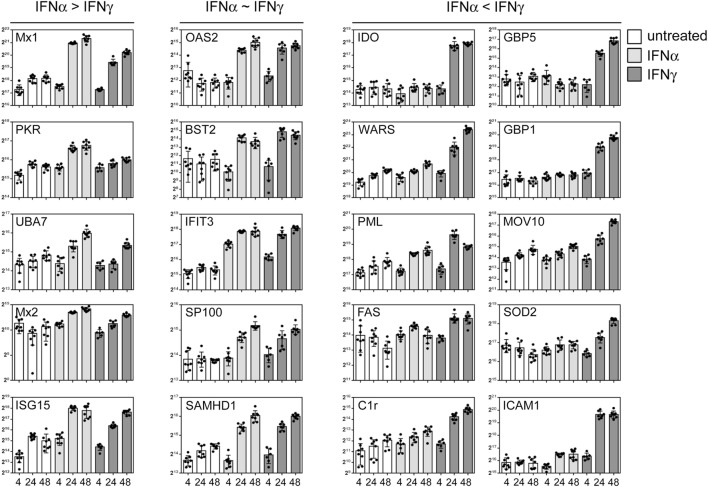
Differential interferon (IFN) responsiveness of effector proteins. Quantifications of indicated proteins are depicted as in Figure 4. The left panel shows selected proteins which are more IFNα responsive. The central panel depicts selected proteins which are similarly IFNα and IFNγ responsive, and the right panel highlights selected proteins which are more responsive to IFNγ. The proteins were chosen according to their previously described role in antiviral activity. The complete set of quantified proteins can be found in the Data Sheet S1 in Supplementary Material.

As outlined above, IFNs elicit pronounced anti-pathogenic activity. However, IFN-I and IFN-II differ in their relative activity against certain taxa of pathogens. Antiviral activity is executed by a multitude of effector proteins. Thus, the discrepancy in efficacy against different agents must also be reflected in the differential expression of the relevant restriction factors. Based on the global data set, we chose prototypic examples of IFN-responsive proteins with documented antipathogenic activity to highlight that certain proteins are similarly responsive to both IFNs, whereas other are either more IFNα or IFNγ inducible. Although some classical antiviral genes such as Mx1 and PKR responded more to IFNα, considerable induction became also evident upon IFNγ treatment (Figure 5, left panel). A second set of proteins with previously shown antiviral activity against viruses such as HIV and influenza [e.g., BST2/Tetherin, SAMHD1, and IFIT3 (40–42)] were similarly responsive to both IFNs (Figure 5, middle panel). Most interestingly, a third class of proteins which was more or even almost exclusively responsive to IFNγ contained proteins such as IDO, GBP5, and PML (Figure 5, right panel) which are known to confer antiviral activity (43–45).

### Early Upregulation of Proteins which become Later Repressed by IFNγ

**Figure 6:**
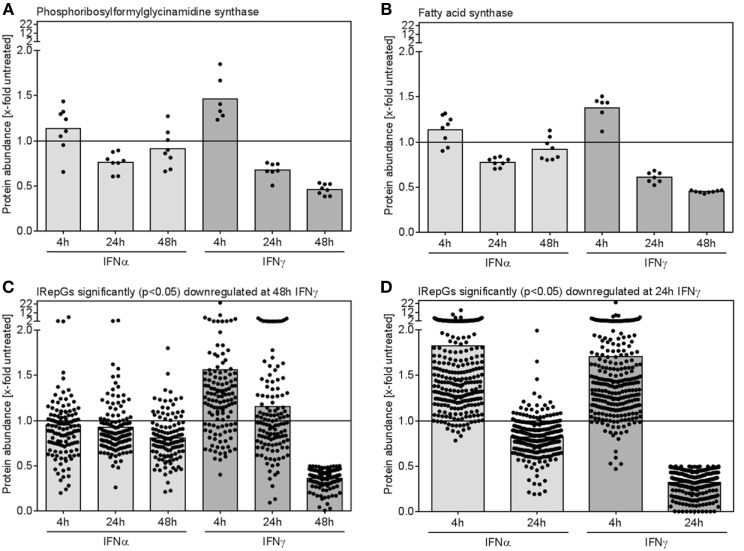
Dynamics of individual and global regulation of interferon (IFN)-repressed proteins. **(A,B)** The relative changes of protein abundance when compared with untreated control cells for two representative IFN-repressed genes (IRepGs) are depicted. **(C)** All proteins significantly (*p* < 0.05) downregulated at 48 h of IFNγ treatment were examined concerning IFNα- and IFNγ-dependent regulation. **(D)** The 4 and 24 h regulation of proteins significantly (*p* < 0.05) downregulated at 24 h of IFNγ treatment is shown. Dots represent individual quantifications **(A,B)** or individual proteins **(C,D)**, bars depict the mean regulation of the respective group.

When IFNγ-repressed proteins were assessed, two interesting trends became apparent: (I) Individual proteins which were significantly repressed after 24 and 48 h of IFNγ exposure showed a similar but less pronounced trend toward a slightly decreased expression upon 24 and 48 h of IFNα treatment (Figures 6A,B). (II) More surprisingly, at early time points of IFNγ conditioning, the same proteins exhibited a trend toward an increase (!) in protein abundance (Figures 6A,B). Both effects also prevailed on global level, when all proteins being significantly downregulated after 48 h (Figure 6C) or after 24 h (Figure 6D) were grouped.

Taken together, the herein described data set uncovers several novel insights into the dynamics and the type specificity of the proteome alterations induced by IFNs, constituting an ideal starting point for mechanistic studies, e.g., into the exact signaling pathways leading to the repression of IRepGs and their biological relevance in defense against pathogens and tumors.

## Discussion

### A Comprehensive Analysis of Type I and Type II IFN-Regulated Proteins

Our analysis establishes a comprehensive catalog of proteins which change their abundance after different time periods of IFN-I and/or IFN-II exposure. Some studies on IFN-induced proteome alterations have been performed previously—usually applying two-dimensional gel electrophoresis and subsequent identification of individual spots being differentially regulated (46, 47). In other studies, only one IFN type, one time point or a specific cellular compartment was assessed (48–50). To our knowledge, we present the first study in which global proteome alteration induced by type I and II IFNs were directly compared in the same cells at different time points. The fact that our data set included six to eight replicates for each condition, the use of the clinically relevant IFNα subtype IFNα2 and a non-transformed cell line frequently used in virus and vaccine research establishes our data set as a reference for future studies. Especially when pathogen- or tumor-induced changes of the cellular proteome are quantified, the induction of IFNs and subsequent IFN-dependent changes are obvious confounding factors. A comparison with the herein described data set will allow others to discriminate IFN-dependent and IFN-independent effects from pathogen-specific changes.

Numerous studies have documented pronounced antipathogenic or antitumor activities of IFNs. However, the actual effector mechanisms are mostly elusive in most cases. The herein described data set establishes an ideal resource and starting point for mechanistic studies on antipathogenic, anti-proliferative, and immune stimulatory effector functions.

### Differential Protein Regulation by IFN-I and IFN-II

The biological responses of IFN types differ. Certain viruses are more susceptible to IFNα, whereas others (e.g., mouse and human cytomegalovirus as well as Vaccinia virus) are more susceptible to IFNγ (20, 38, 51). This discrepancy is highlighted by the fact that individual IFNs are approved for the treatment of defined diseases, whereas other IFNs are not. Based on the wealth of information on cross-talk between the signaling cascades and the overlapping responsiveness of certain ISGs, this biological difference was rather puzzling. However, our comparative analysis of the global changes within proteomes is fully consistent with this discrepancy: in terms of quality, quantity, and kinetics, IFN-induced changes differ significantly between IFN-I and IFN-II. This effect was most apparent for repressed proteins which were almost exclusively observed in response to IFNγ.

Interestingly, although our data uncovers remarkable differences between IFN classes and incubation times, the simple dichotomy that ISRE-activating IFNs (types I and III) act directly antiviral, whereas the GAS-activating IFNγ acts immune-modulatory is clearly oversimplified: IFNγ induces several direct antiviral effector proteins at least as strong as IFNα or even stronger. Additionally, IFNα also enhances the components of MHC presentation.

The biological response to IFNs can be influenced by the abundances of the corresponding IFN receptors. Differential responses toward IFNα when compared with IFNγ might be influenced by different receptor surface dispositions. Surface levels of proteins can be determined by cytometry using antibodies (or ligands) coupled to fluorophores. However, these antibodies recognize their cognate antigens with different affinities resulting in an unavoidable bias in the comparative quantification of different proteins. Similarly, MS-based determinations rely on different peptides with non-overlapping characteristics during identification and quantification. For our analysis, we used saturating IFN concentrations. Depending on the ISG and IRepG subsets, we observed pronounced responses either to IFNα (see Figure 5, left panel), to IFNγ (Figure 5, right panel) or both IFNs (Figure 5, central panel) indicating the perceptiveness of the cells. In addition, our finding that the differential responses (e.g., gene repression of IRepGs stimulated by IFNγ but not by IFNα) were observed on different levels (protein and mRNA), in different species (mouse and human) and in different cell types (macrophages and fibroblasts) suggest that the herein described differences are rather common. However, it might be that certain cell types with heavily skewed expression levels of the IFN receptor complexes might depict altered responses.

### Existence of IFNγ-Repressed Proteins in Human Cells

Our previous transcriptional analysis in murine cells revealed the existence of an extended set of IRepGs in mouse fibroblasts and macrophages (22). IRepG responses constitute a primary transcriptional response resistant to the blockade of translation by cycloheximide. An alignment of IRepGs and their promoters/enhancers did not result in an enrichment of GAS or ISRE elements but instead uncovered an accumulation of GC-rich elements and corresponding SP1/SP3 binding sites (22). This was consistent with previously described SP1/SP3 dependency of individual genes shown to be repressed by IFNγ (52). Despite the lack of ISRE and GAS sequences, IRepG repression was largely lost in STAT1-deficient fibroblasts, suggesting that gene repression by IFNγ is a mostly neglected ability of its “signature” transcription factor STAT1. The data immediately raised two important questions: (I) Do IRepGs exist in other species and especially in cells of human origin and (II) does IFNγ-induced transcriptional gene repression translate into reduced abundance of proteins? This study provides answers to both questions: we observed a surprisingly large number of proteins being negatively regulated by IFN and especially type II IFN in human cells: after 4 h of treatment, the number of IFNγ-repressed proteins even exceeded the number of induced proteins. At 24 and 48 h post treatment, approximately two ISG proteins face one IRepG protein. The top hits of our global analysis include proteins such as collagen α1 and 2 for which individual experiments have documented repression by IFNγ (53, 54) further validating our data set in regard to the IRepGs.

This poses the important future question concerning the biological significance(s) of these IRepGs in terms of the response to pathogens and tumors. It is not difficult to imagine that the IFN-induced downregulation of proteins which are required for the replication of certain pathogens or tumors might have benefits for the host—simply by withholding essential cofactors, e.g., the entry receptor of an intracellular pathogen to prevent its infection. Viruses also rely on numerous intracellular factors and metabolic capabilities of the host cell to allow their own replication. One unifying feature of viruses is their lack of ribosomes and thus their inability to translate proteins outside of host cells. Consistently, the translational machinery is one major battleground between pathogens and host, and several ISGs (e.g., PKR, ISG54, and ISG56) target translation [reviewed in Ref. (55)]. A downregulation of proteins required for translation (or other essential metabolic pathways such as ATP synthesis) might limit the replication of pathogens. Consistently, we observed that, e.g., eIF2A, eIF3M, and others were repressed by IFNγ (see Data Sheet S1 in Supplementary Material). Thus, it seems that IRepGs contribute to the restriction of the translational machinery. The seemingly suicidal aspect of such repression might be overcome by strictly controlled (down-)regulation by IFNs upon infection.

The question arises if we can find defined host factors amongst the IFN-repressed proteins? One protein which was downregulated is fatty acid synthase FASN (protein ID P49327, Figure 6B). From different studies, FASN is known to be required for the replication of hepatitis C virus, dengue virus, respiratory syncytial virus, human parainfluenza 3 (PIV3), astrovirus, and rhinovirus replication (56–60). Therefore, it is tempting to speculate that suppression of FASN by IFNγ might reduce the replication of such viruses. Another example is the protein Ergic53 which is required for the replication of Arena, Corona, and Filoviruses (61), and which we found to be repressed by IFNγ. Functional studies on IRepGs are definitely needed, but a comparison of herein found IRepGs with genes found in at least two of the three published siRNA screens to be required for HIV replication (62) indicate that the family of IRepGs also comprises additional genes required for HIV replication (e.g., IDH1).

Another protein which was significantly downregulated was PFDN1 (protein ID O60925). Interestingly, PFDN1 has recently been shown to promote epithelial-mesenchymal transition and lung cancer progression (63). Therefore, it is also tempting to speculate that this—or similar effects—of protein repression might also contribute to the antitumor effects of IFN. However, it remains to be elucidated in the future if, to which extent, and by which mechanisms IRepGs exactly contribute to known properties such as antiviral, antibacterial, antiproliferative, antitumor, and immune-stimulatory effects of IFN or maybe to the side-effects induced by IFN treatment.

### Early IFN-Induced Expression of IRepGs

The finding that IFN-repressed proteins significantly downregulated upon 24 and 48 h of IFNγ treatment showed slightly increased abundances after 4 h of IFN is at first glance challenging to reconcile. Future studies are required to elucidate the involved molecular events and transcription factors leading to gene repression by IFN. A very interesting future question in this regard is the contribution of the transcriptional changes in comparison to altered protein stability.

Taking our previous findings that IRepGs can be observed on the level of nascent mRNA and that the effect is largely STAT1 dependent (22) as well as the herein described early induction into account, a potential explanation might be based on the negative feedback regulation of IFNs. It is well known that IFNs limit their own signaling by stimulating potent mediators of negative feedback loops (e.g., IFN-induced expression of suppressors of cytokine signaling and protein inhibitor of activated STATs). We hypothesize that IRepGs might be weakly IFN responsive but fully responsive to the effects of the negative feedback cascade. We infer that the responsible proteins should be (I) repressors of transcription, (II) IFN-inducible, and (III) able to “identify” IRepGs. Beside SP1 and SP3, three promising candidates for such mechanisms are IRF-2, PRDM1, and Bcl6. All three proteins bind to DNA and repress transcription (64–67), all are induced by IFN and recognize ISRE, IRF-E, GAS, or similar DNA elements (30, 65, 68, 69).

Taken together, our analysis provides a comprehensive catalog of IFN-induced changes of the human proteome. Remarkable differences in terms of the proteomic changes induced by IFNα and IFNγ became evident—especially upon prolonged exposure. The proteomes of IFN-stimulated cells change dramatically during the duration of IFN stimulation, highlighting the necessity to perform experiments in which biological responses are compared with protein expression data with adequate temporal resolution and comparability. Additionally, a mostly neglected class of genes/proteins being significantly repressed by IFN—and IFNγ in particular—was found to exist on the level of the human proteome.

## Author Contributions

DM, JP, and VTKL-T conducted the experiments. All authors analyzed the data. DM, BS, and MT designed the experiments and supervised the project. DM and MT wrote the article.

## Conflict of Interest Statement

The authors declare that the research was conducted in the absence of any commercial or financial relationships that could be construed as a potential conflict of interest.
