# Supplementary material for: Deciphering of the Human Interferon-Regulated Proteome by Mass Spectrometry-Based Quantitative Analysis Reveals Extent and Dynamics of Protein Induction and Repression
Source: Front Immunol. 2017 Sep 14;8:1139. doi: 10.3389/fimmu.2017.01139 (PMC5603615; doi:10.3389/fimmu.2017.01139)
Supplement: Supplementary file 3 [file Data_Sheet_1.DOCX]

Deciphering the human interferome and its kinetics of protein induction and repression by mass spectrometry-based quantitative proteome analysis

Supplemental material

Content:

1. Table S1: Overview of LC-MS/MS identifiers.

2. Figure S1: Accuracy of protein quantification.

3. Figure S2-S5: Functional enrichment analysis of regulated proteins.

4. Supplemental file 1: Excel-file containing the list of 2,735 quantified proteins.

5. Supplemental file 2: Excel-file containing the results of enrichment analyses.

Table S1. Overview of LC-MS/MS runs analyzed in the proteomics study. Using the respective sample identifiers, deposited mass spectrometric data can be assigned to the investigated experimental groups.

| **LC-MS/MS run** | **Treatment** | **Time** |
| --- | --- | --- |
| OEI13507 | IFNα | 24 h |
| OEI13528 | IFNα | 24 h |
| OEI13540 | IFNα | 24 h |
| OEI13546 | IFNα | 24 h |
| OEI13582 | IFNα | 24 h |
| OEI13591 | IFNα | 24 h |
| OEI13631 | IFNα | 24 h |
| OEI13667 | IFNα | 24 h |
| OEI13509 | IFNα | 48 h |
| OEI13511 | IFNα | 48 h |
| OEI13517 | IFNα | 48 h |
| OEI13548 | IFNα | 48 h |
| OEI13558 | IFNα | 48 h |
| OEI13574 | IFNα | 48 h |
| OEI13599 | IFNα | 48 h |
| OEI13609 | IFNα | 48 h |
| OEI13515 | IFNα | 4 h |
| OEI13536 | IFNα | 4 h |
| OEI13552 | IFNα | 4 h |
| OEI13566 | IFNα | 4 h |
| OEI13605 | IFNα | 4 h |
| OEI13627 | IFNα | 4 h |
| OEI13645 | IFNα | 4 h |
| OEI13647 | IFNα | 4 h |
| OEI13556 | IFNγ | 24 h |
| OEI13568 | IFNγ | 24 h |
| OEI13580 | IFNγ | 24 h |
| OEI13593 | IFNγ | 24 h |
| OEI13621 | IFNγ | 24 h |
| OEI13637 | IFNγ | 24 h |
| OEI13663 | IFNγ | 24 h |
| OEI13530 | IFNγ | 48 h |
| OEI13554 | IFNγ | 48 h |
| OEI13576 | IFNγ | 48 h |
| OEI13578 | IFNγ | 48 h |
| OEI13595 | IFNγ | 48 h |
| OEI13623 | IFNγ | 48 h |
| OEI13649 | IFNγ | 48 h |
| OEI13653 | IFNγ | 48 h |
| OEI13538 | IFNγ | 4 h |
| OEI13570 | IFNγ | 4 h |
| OEI13611 | IFNγ | 4 h |
| OEI13613 | IFNγ | 4 h |
| OEI13657 | IFNγ | 4 h |
| OEI13661 | IFNγ | 4 h |
| OEI13513 | Mock | 24 h |
| OEI13520 | Mock | 24 h |
| OEI13522 | Mock | 24 h |
| OEI13544 | Mock | 24 h |
| OEI13572 | Mock | 24 h |
| OEI13617 | Mock | 24 h |
| OEI13619 | Mock | 24 h |
| OEI13629 | Mock | 24 h |
| OEI13526 | Mock | 48 h |
| OEI13534 | Mock | 48 h |
| OEI13562 | Mock | 48 h |
| OEI13564 | Mock | 48 h |
| OEI13607 | Mock | 48 h |
| OEI13625 | Mock | 48 h |
| OEI13651 | Mock | 48 h |
| OEI13671 | Mock | 48 h |
| OEI13597 | Mock | 4 h |
| OEI13601 | Mock | 4 h |
| OEI13603 | Mock | 4 h |
| OEI13635 | Mock | 4 h |
| OEI13639 | Mock | 4 h |
| OEI13641 | Mock | 4 h |
| OEI13655 | Mock | 4 h |
| OEI13665 | Mock | 4 h |





Figure S1. Coefficient of variation (CV) of protein quantifications depending on the number of unique peptides used for quantification. The exclusion of 210 single peptide quantifications with large deviations as indicated by an averaged CV >50% led to a decreased variation, comparable to the one observed for proteins quantified with two peptides.





Figure S2. Enrichment analysis using DAVID (Ver. 6.8) based on GO annotations of proteins being significantly up- or down-regulated (p < 0.05, at least twofold regulation) by IFNα after different times of exposure. Numbers of annotated proteins are shown as bars color-coded regarding the categories biological process (white), cellular component (light grey) and molecular function (grey). Significances are indicated as dot-line plots of -log10-transformed p-value (Benjamini-Hochberg corrected values).





Figure S3. Enrichment analysis using DAVID (Ver. 6.8) based on GO annotations of proteins being significantly up- or down-regulated (p < 0.05, at least twofold regulation) by IFNγ after 4 hours. Numbers of annotated proteins are shown as bars color-coded regarding the categories biological process (white), cellular component (light grey) and molecular function (grey). Significances are indicated as dot-line plots of -log10-transformed p-value (Benjamini-Hochberg corrected values).





Figure S4. Enrichment analysis using DAVID (Ver. 6.8) based on GO annotations of proteins being significantly up- or down-regulated (p < 0.05, at least twofold regulation) by IFNγ after 24 hours. Numbers of annotated proteins are shown as bars color-coded regarding the categories biological process (white), cellular component (light grey) and molecular function (grey). Significances are indicated as dot-line plots of -log10-transformed p-value (Benjamini-Hochberg corrected values).





Figure S5. Enrichment analysis using DAVID (Ver. 6.8) based on GO annotations of proteins being significantly up- or down-regulated (p < 0.05, at least twofold regulation) by IFNγ after 48 hours. Numbers of annotated proteins are shown as bars color-coded regarding the categories biological process (white), cellular component (light grey) and molecular function (grey). Significances are indicated as dot-line plots of -log10-transformed p-value (Benjamini-Hochberg corrected values).
